# Editorial: Single membrane channels formed by connexins or pannexins: focus on the nervous system

**DOI:** 10.3389/fncel.2015.00402

**Published:** 2015-10-15

**Authors:** Juan A. Orellana

**Affiliations:** Departamento de Neurología, Escuela de Medicina, Pontificia Universidad Católica de ChileSantiago, Chile

**Keywords:** connexins, brain, microglia, astrocyte, neuron, pannexin, hemichannels

For many years, the main function attributed to connexin hemichannels was providing the building blocks of gap junctions channels (GJCs), which allow direct but selective cytoplasmic continuity and molecular exchange between contacting cells. Nonetheless, the presence of functional connexin hemichannels in “nonjunctional” membranes has been demonstrated by several experimental approaches. These channels act like aqueous pores that are permeable to ions and small molecules and thus provide a diffusional route of exchange between the intra- and extracellular milieu. Recently, another gene family encoding a set of three membrane proteins termed pannexins (Panxs 1–3) was identified. Currently, most of evidence indicates that pannexins support the formation of single membrane channels (pannexons), similar, to connexin hemichannels; permitting paracrine/autocrine signaling among cells.

Paracrine signaling mediated by connexin hemichannels and pannexons is emerging as one of the most widely distributed mechanisms of synchronization in the physiological brain parenchyma. However, it is believed that impairments of the permeability properties of connexin hemichannels and pannexons might be critical to the initiation and maintenance of the homeostatic imbalances that are observed in diverse brain diseases. In this collection, we gather a wide collection of 20 original research and review articles, providing the latest progress and insights in the field of connexin hemichannels and pannexons in the nervous system.

Although, about 50% of autosomal recessive non-syndromic hearing loss occur by connexin mutations, the involvement of connexins in the etiology of acquired hearing loss remains to be fully elucidated. In this context, Figueroa and colleagues shed light on the gentamicin-induced inhibition of Cx26 hemichannels as possible cause of post-lingual hearing loss evoked by this aminoglycoside antibiotic (Figueroa et al., 2014). A puzzling aspect of connexin and pannexin field is found pharmacological tools allowing to distinguish between the function of hemichannels vs. GJCs. By employing primary cultures as well as acute hippocampal slices, Abudara and collaborators show that Gap19, a nonapeptide derived from the cytoplasmic loop of Cx43, inhibits astrocytic Cx43 hemichannels in a dose-dependent manner, without affecting GJCs (Abudara et al., 2014). This first section is closed with an article that examines the trafficking and subcellular localization of endogenous Panx2 and Panx1 proteins in astrocytes and neurons (Boassa et al., 2014), whereas the last study demonstrates that endogenous expression of Panx2 protein is not exclusively restricted to the nervous system (Le Vasseur et al., 2014).

The next section start with an elegant review addressing how hemichannel composition and intercellular gradient of charged cytosolic factors determines the symmetry and rectification of electrical transmission (Palacios-Prado et al., 2014). “Connexons and pannexons: newcomers in neurophysiology” by Cheung et al., reviews the involvement of connexons and pannexons in synaptic transmission and behavior. They summarize current knowledge about how connexin hemichannels and pannexin channels are involve in neuronal excitability, synaptic transmission, learning, and memory, providing as well an outlook on whether these channels could exhibit cell-type specific regulations or even release different combinations of molecules under varying circumstances (Cheung et al., 2014). “Pannexin 1 regulates bidirectional hippocampal synaptic plasticity in adult mice” by Ardiles and colleagues, proposes that pharmacological or genetic ablation of Panx1 enhance synaptic transmission by reducing extracellular levels of ATP in the synaptic cleft (Ardiles et al., 2014). The role of Panx1 channels in transmitter release is not confined to the central nervous system. Indeed, Momboisse and collaborators provide pioneering data supporting that Panx1 channels contribute to the exocytotic release of catecholamines in chromaffin cells (Momboisse et al., 2014).

Further, diverse articles and reviews analyze the involvement of connexin hemichannels and pannexins channels in different sensory cells and systems. “Investigation of olfactory function in a Panx1 knock out mouse model” proposes that although Panx1 channels contribute to the ATP release in the olfactory epithelium, characterization of Panx1^−∕−^ mice does not support a prominent role of Panx1 in olfaction (Kurtenbach et al., 2014b). Kurtenbach and colleagues also review in “Emerging functions of pannexin 1 in the eye” how Panx1 is involved in processing visual information, as well as its role in different pathological conditions such as hypoosmotic stress and glaucoma (Kurtenbach et al., 2014a). Despite that accumulating evidence has shown that connexin-based channels are involve in retinal neural coding in nocturnal rodents, the contribution of these channels to signal processing in the retina of diurnal rodents is still unclear. The research article “Role of connexin channels in the retinal light response of a diurnal rodent” by Palacios-Muñoz and colleagues, deals with this matter and by using *in vivo* ERG recording under scotopic and photopic light adaptation, they examine the contribution of connexin-based channels to the retinal light response in the diurnal rodent *Octodon degus* compared to rat (Palacios-Muñoz et al., 2014). In “Opening of pannexin- and connexin-based channels increases the excitability of nodose ganglion sensory neurons,” Retamal and colleagues show that divalent cation-free solution, a condition that enhance connexin hemichannel opening, increases the electrical activity of vagal nerve by a mechanism that depend on hemichannels, Panx1 channels and P2X_7_ receptors (Retamal et al., 2014). In closing this section, “Cxs and Panx- hemichannels in peripheral and central chemosensing in mammals” by Reyes et al., provides novel information on participation of connexons and pannexons in arterial and central chemoreception (Reyes et al., 2014).

At the beginning of the last section, “Neuronal involvement in muscular atrophy” by Cisterna et al., discusses the potential role of relevant factors in maintaining the physiological functioning of fast skeletal muscles and suppression of hemichannel expression (Cisterna et al., 2014). Mutations in Cx26, are the most usual causes of hereditary, sensorineural hearing loss. “Aberrant Cx26 hemichannels and keratitis-ichthyosis-deafness syndrome: insights into syndromic hearing loss” by Sanchez and Verselis, summarizes some of the aberrant Cx26 hemichannel properties that have been reported for mutants associated with keratitis-ichthyosis-deafness (KID) syndrome, a particularly severe Cx26-associated syndrome. They advocate for exploring and elucidate genotype-phenotype relationships and causes underlying cochlear dysfunction (Sanchez and Verselis, 2014). In closing, this e-book provides a nice overview that shed lights in new aspects on the role of connexons and pannexons in the nervous system during neurodegenerative and inflammatory conditions. “Regulation of gap junction channels by infectious agents and inflammation in the CNS” by Castellano and Eugenin, discusses recent findings regarding the critical role of GJCs in the pathogenesis of brain infectious diseases and associated inflammation (Castellano and Eugenin, 2014). The possible consequences of chronic hemichannel opening in neurodegenerative disorders, particularly, Alzheimer's disease (AD) and lysosomal storage disorders, are highlighted and discussed in “Hemichannels in neurodegenerative diseases: is there a link to pathology?” (Bosch and Kielian, 2014). Meanwhile, Takeuchi and Suzumura provide mechanistic insight on how the release of glutamate through hemichannels from microglia could affect neuronal survival in different brain pathologies, including AD, stroke, multiple sclerosis and amyotrophic lateral sclerosis (Takeuchi and Suzumura, 2014). In “Prenatal nicotine exposure enhances Cx43 and Panx1 unopposed channel activity in brain cells of adult offspring mice fed a high-fat/cholesterol diet,” Orellana and collaborators investigate how prenatal (nicotine) and postnatal (high fat/cholesterol diet) stimuli increase the opening of connexons and pannexons in brain cells of adult mice (Orellana et al., 2014). Pioneering findings by Orellana and colleagues also demonstrate that chronic restraint stress increases the opening of hemichannels and pannexin channels in the brain. They propose that gliotransmitter release through connexons and pannexons may participate in the pathogenesis of stress-associated psychiatric disorders and possibly depression (Orellana et al., 2015).

A growing body of evidence supports the notion that hemichannels and pannexons seem to be active under physiological conditions. Apparently, these channels exhibit a low activity in normal than in pathological states, but they are sufficiently open to ensure cellular signaling in the nervous system. Do the permeability properties of hemichannels remain unaltered during neurodegeneration? How do changes in the permeabilities of hemichannels to Ca^2+^ and different gliotransmitters influence brain diseases? Which posttranslational modifications are responsible of these changes? These are some of the puzzling problems that the upcoming studies should try to address. Characterization of the fundamental elements that specifically regulate connexin and pannexin function in physiological and pathophysiological conditions will enable the identification of future therapies for neurological disorders.

## Conflict of interest statement

The author declares that the research was conducted in the absence of any commercial or financial relationships that could be construed as a potential conflict of interest.
